# Intimate partner violence and psychological interventions in low-income and middle-income countries

**DOI:** 10.1016/S2215-0366(19)30530-9

**Published:** 2020-02

**Authors:** Obianuju O Berry, Catherine Monk

**Affiliations:** Department of Psychiatry (OOB, CM) and Department of Obstetrics and Gynecology (CM), Columbia University Medical Center, New York, NY, USA; and New York State Psychiatric Institute, New York, NY 10032, USA (OOB, CM)

Intimate partner violence is widespread and prevalent, with the WHO identifying it as a global health problem of epidemic proportions affecting high-income countries and low-income and middle-income countries (LMICs) at prevalences ranging from 27·9% to 45·6%.^1^ Although men also are victims, intimate partner violence is considered gender-based violence owing to differences in the severity, frequency, type, and lifetime impact of intimate partner violence on women. Despite the high prevalence of intimate partner violence in society and the strong, bidirectional association between intimate partner violence and mental health disorders, systematic and large-scale randomised treatment trials that include indices of intimate partner violence are rare, restricting opportunities for the translation of clinical research into evidence-based practice for those exposed to intimate partner violence.

The systematic review and meta-analysis by Roxanne C Keynejad and colleagues^2^ in *The Lancet Psychiatry* has many conceptual strengths and innovative aspects that greatly contribute to its public health relevance, high impact, and novelty. The authors focus on psychological treatment studies in LMICs where emerging evidence supports brief interventions for common mental disorders (CMDs).^3^ This analysis is the first exploration of intimate partner violence as a moderator of psychological treatment effectiveness for CMDs. Previous research on psychological treatment in LMICs has addressed implementation barriers, such as limited resources, task sharing, uptake of a proven intervention, or patient and clinician barriers.^4^ For mental health disorders, Keynejad and colleagues consider CMDs as a whole as opposed to only one diagnostic classification. There is wide heterogeneity in disorders associated with intimate partner violence, with the most prevalent being depression, anxiety, post-traumatic stress disorder (PTSD), and substance use disorders.^5^ However, intimate partner violence is also associated with subsyndromal symptoms of mental health disorders, such as psychological distress symptoms, which were an outcome in this study.

Keynejad and colleagues screened over 5000 records of 8122 initially identified; of the 491 full-text records eligible for inclusion on the first pass of screening (abstract or title), only 96 (20%) measured intimate partner violence exposure. Ultimately, 15 randomised controlled trials were included from eight LMICs (Kenya, South Africa, Zimbabwe, Uganda, Cambodia, Pakistan, India, and Iraq), enabling the authors to generate enough statistical power to address intimate partner violence moderation effects on treatment outcomes of psychological interventions. Numbers of sessions ranged from three to 14 delivered at clinics, in community settings, at home, or a mixture of locations. Most interventions were delivered individually.

Contradicting the commonly held dogma that non-tailored treatments will be ineffective for those with intimate partner violence, results suggested that women who had experienced intimate partner violence versus those who had not might benefit more from psychotherapies, even when the treatment does not include specially targeted components for intimate partner violence. This finding was especially pronounced for anxiety, such that there was greater improvement in anxiety symptoms in the context of intimate partner violence. Similar findings were seen for depression, PTSD, and general distress, but the differences were not statistically significant. These results raise important questions about potentially unique psychological and neurobiological characteristics of anxiety in the context of trauma. The broader study finding is that none of the interventions specifically addressed intimate partner violence or CMD symptoms arising from intimate partner violence, yet intimate partner violence survivors with CMDs benefited from the generic treatments. Specifically, comparing trauma-focused interventions with more basic behavioral activation and cognitive behavioral therapy approaches did not show differences in the moderation effect of intimate partner violence on treatment outcomes.

Childhood trauma and the role of polyvictimisation were outside the scope of the study. Women with histories of childhood maltreatment are more likely to suffer from intimate partner violence and from CMDs.^6,7^ Several studies^8,9^ indicate that childhood maltreatment can adversely affect intervention outcomes, resulting in longer times to remit, preference for one psychotherapy over the other, and less responsiveness to psychopharmacology. Could it be that those women with histories of intimate partner violence and depression, PTSD, or psychological distress, did not show enhanced improvement with psychological interventions due, in part, to histories of childhood maltreatment? More research is needed to answer this question.

Finally, because the majority of women are mothers or will go on to parent, the psychological sequelae of intimate partner violence could include adverse effects on attachment schemas and associated caregiving abilities and the capacity to foster trusting relationships with their children. Future research on intimate partner violence treatment could usefully consider aspects of the mother–child relationship to make a contribution towards diminishing intergenerational trauma effects.

Intimate partner violence is common and its effects on women’s health can be profound. The paucity of evidence for psychological interventions tailored to the experiences of women affected by intimate partner violence means that it is unclear whether specialised, trauma-informed treatments in LMICs would be more effective than generic interventions for CMDs. Incorporating analysis of sex and gender into research—well illustrated by the inclusion of intimate partner violence as a treatment moderator—can foster discovery, increase experimental reproducibility, and promote social equity.^10^
